# Utility and Predictive Value of Human Standard Semen Parameters and Sperm DNA Dispersion for Fertility Potential

**DOI:** 10.3390/ijerph16112004

**Published:** 2019-06-05

**Authors:** Kamil Gill, Joanna Jakubik, Aleksandra Rosiak-Gill, Michał Kups, Mariusz Lukaszuk, Maciej Kurpisz, Monika Fraczek, Małgorzata Piasecka

**Affiliations:** 1 Department of Histology and Developmental Biology, Pomeranian Medical University in Szczecin, 71-210 Szczecin, Poland; kamilgill@wp.pl (K.G.); jakubik_joanna@wp.pl (J.J.); rosiak.aleksandra@yahoo.com (A.R.-G.); michalkups1@gmail.com (M.K.); 2 VitroLive Fertility Clinic in Szczecin, 70-483 Szczecin, Poland; 3 Department of Urology and Oncological Urology, Regional Specialist Hospital in Szczecin, 71-455 Szczecin, Poland; 4 Healthcare Center Nowe Orlowo, 81-525 Gdynia, Poland; m.lukaszuk@gumed.edu.pl; 5 Invicta Fertility Clinic, 80-850 Gdansk, Poland; 6 Institute of Human Genetics, Polish Academy of Sciences, 60-479 Poznan, Poland; maciej.kurpisz@igcz.poznan.pl (M.K.); monika.fraczek@igcz.poznan.pl (M.F.)

**Keywords:** semen analysis, male fertility potential, sperm DNA fragmentation

## Abstract

Because the assessment of sperm DNA fragmentation (SDF) plays a key role in male fertility, our study was designed to find the relationships between SDF and standard semen parameters. The receiver operating characteristic (ROC) curve showed that 18% SDF is a prognostic parameter for discriminating between men with normal and abnormal standard semen parameters (n = 667). Men with > 18% SDF had significantly lower quality semen, a higher prevalence of abnormal semen characteristics, and a higher odds ratio for abnormal semen parameters compared to men with ≤ 18% SDF. An ROC analysis provided predictive values for age and semen parameters to distinguish between men with SDF > 18% and men with ≤ 18% SDF. SDF was positively correlated with male age and teratozoospermia index but negatively with sperm concentration, total number of spermatozoa, sperm morphology, progressive motility, and vitality. Our study shows that 18% SDF has a predictive value for distinguishing between men with normal and abnormal semen characteristics. Men with >18% SDF have a higher risk for abnormal semen parameters, while age and obtained semen parameters have a predictive value for SDF. There is a relationship between SDF and conventional sperm characteristics, and thus, SDF can be incorporated into male fertility assessment.

## 1. Introduction

Approximately up to 20% of couples trying to achieve pregnancy suffer from infertility [1,2,3]. It is known that male factors are responsible for 20–70% of cases, and one-third of these cases may be caused by male factors alone [1,2,3,4,5]. Male factors can influence not only the fertilization process but also embryo gene expression and development. In addition, male factors may also be involved in idiopathic miscarriages, as well as autosomal dominant diseases and neurobehavioural disorders in offspring, especially in cases of advanced paternal age [6,7,8,9,10]. Commonly, the assessment of male fertility potential is based on standard semen analysis. However, evidence from recent years has shown that basic seminological analysis may not always be an optimal diagnostic tool, but it still remains the basic clinical tool for evaluation male fertility potential [11,12]. On the other hand, some reports have even indicated the limited clinical utility of this analysis and demonstrated that searching for the best biomarker for the diagnosis of male infertility is required [13,14,15,16,17,18]. 

Many authors have shown that knowledge about the level of DNA damage is essential and provides the possibility of an optimal approach to an infertility problem [13,18,19,20,21]. Sperm DNA fragmentation is a male infertility factor associated with failure to conceive, longer times to pregnancy, poor outcome of infertility treatment (including in vitro fertilization), impaired embryo development, higher miscarriage rates, and health problems in offspring [13,15,21,22,23]. It should be highlighted that clinical data show that when the SDF is above 20%, the chance of natural pregnancy may be reduced, and that when SDF is above 30%, the chance for achieving pregnancy in natural conception or by insemination is very low [11,12,24,25,26,27,28]. For this reason, an analysis of SDF is recommended, particularly in difficult clinical cases, such as varicocele (often before varicocelectomy), idiopathic male infertility, miscarriages, unsuccessful ART treatment, influence of an unhealthy lifestyle, and advanced paternal age [13,19,20]. Therefore, the aims of our study were to 1) estimate the threshold of SDF distinguishing males with abnormal standard semen parameters (decreased fertility potential) and normal standard semen parameters, and 2) perform a mutual analysis of associations between sperm DNA fragmentation, age and standard semen parameters. 

## 2. Subjects

The study was performed on ejaculated sperm cells obtained from a general population of men (n = 667, median of age = 32 years) attending the Andrology Laboratory of Department of Histology and Developmental Biology (Pomeranian Medical University in Szczecin, Poland). The laboratory is a research and scientific unit and access to research was open and unlimited—any adult man could take part in the research. The groups of men with normal standard semen parameters (n = 234) and with abnormal standard semen parameters (n = 434) were created according to the World Health Organization (WHO) [29] criteria. In both groups, the exclusion criteria included the following: a clinical picture suggestive of obstructive azoospermia, a history of testicular torsion, maldescent, injury or cancer, co-existing systemic disease, and a history of mumps. The ethics committee of the Pomeranian Medical University, Szczecin, Poland approved the study protocol (ethical authorization number: KB-0012/21/18).

The semen samples were collected after 2–7 days of sexual abstinence by masturbation. Sperm concentration was calculated in an improved Neubauer haemocytometer (Heinz Hernez Medizinalbedarf GmbH, Hamburg, Germany). In turn, sperm motility (total and progressive motility), vitality (live sperm cells: eosin-negative or hypo-osmotic-reactive sperm cells (HOS) test) were examined with phase-contrast microscope (Primo Star, Zeiss, Germany), but sperm morphology with a bright light microscope (CX 31 Olympus Optical Co., Ltd., Tokyo, Japan), respectively. Papanicolaou-stained spermatozoa were used for sperm morphology and teratozoospermia index (TZI) assessment. The concentration of leukocytes in the semen samples (peroxidase-positive cells) was calculated using the Endtz test (LeucoScreen kit, FertiPro N.V., Beernem, Belgium).

Normozoospermia (n = 242) was considered according to the following WHO [29] criteria: sperm concentration ≥15 mln/mL, total number of sperm cells ≥39 mln, sperm progressive motility ≥32% and morphology ≥4%. Furthermore, the TZI, vitality, and concentration of peroxidase-positive cells (leukocytes) were evaluated. In the group of men with abnormal standard semen parameters (n = 434), the following seminological categories were noted: asthenozoospermia (men with abnormal sperm motility, n = 7); oligozoospermia (men with abnormal number of sperm cells, n = 19); teratozoospermia (men with abnormal sperm morphology n = 162); asthenoteratozoospermia (men with abnormal sperm motility and morphology, n = 56); oligoasthenospemia (men with abnormal number and motility of sperm cells, n = 2); oligoteratozoospermia (men with abnormal number and morphology of sperm cells, n = 81); and oligoasthenoteratozoospermia (men with abnormal number, motility and morphology of sperm cells, n = 107).

### 2.1. Sperm Chromatin Dispersion (SCD) Test (Halosperm Test)

The SCD test was used to verify SDF. The evaluation of DNA dispersion after denaturation was carried out using a Halosperm G2 kit (Halotech DNA, Madrid, Spain) following the manufacturer’s guidelines: (1) preparation of a mixture containing sperm cells (≤ 20 mln/mL) and melted agarose (1:2); (2) placement of the sperm suspension (10 µL) on the centre of a super-coated slide; and (3) denaturation, lysis, dehydration, and staining of sperm cells with eosin and thiazine.

The smears were evaluated under a bright light microscope at x1000 magnification (CX 31 Olympus Optical Co., Ltd., Tokyo, Japan). A minimum of 300 spermatozoa per sample were counted. Sperm cells without SDF can produce the characteristic halo of dispersed DNA loops (large halo: halo width similar to or higher than the diameter of the sperm head; medium halo: halo width > 1/3 the diameter of the sperm head), while spermatozoa with damaged DNA fail to form a halo of dispersed DNA loops (small halo: halo width ≤ 1/3 the diameter of the sperm head; sperm cells without a halo or degraded: spermatozoa with no halo or irregular, weakly stained sperm head). The results are presented as the total number of spermatozoa with small or no halo, that is, degraded, divided by the total number of assessed sperm cells, and multiplied by 100% [30,31]. 

### 2.2. Statistical Analyses

The statistical analyses were performed using the software Statistica version 13.3 (StatSoft, Cracow, Poland) and MedCalc version 15.2.2 (MedCalc Software, Ostend, Belgium), with significance set at *p* < 0.05. The quantitative variables are expressed as the mean ± standard deviation (SD) and median (range), while categorical data are reported as percentages. The conformity of numerical variables with the normal distribution was examined using the Shapiro–Wilk test. Therefore, the nonparametric Mann–Whitney U test was used to compare data from two independent groups. A chi-square test was performed to compare the categorical data. The interdependences of the variables were examined by calculating the rank Spearman correlation coefficient (r_s_). To interpret the strength dependence between the study parameters, the following levels of correlation were presumed: <0.2—lack of linear dependence, 0.2–0.4—weak dependence, >0.4–0.7—moderate dependence, >0.7–0.9—strong dependence, >0.9—very strong dependence. The predictive values of obtained parameters were verified using the receiver operating characteristic (ROC) curve and the area under curve (AUC), taking into account the standard error (SE) and 95% confidence interval (CI 95%). The following levels of AUC were: 0.9–1.0—excellent predictive value, >0.8–0.9—good predictive value, >0.7–0.8—satisfactory predictive value, >0.6–0.7—moderate predictive value, 0.5–0.6—insufficient predictive value. The odds ratios (OR) for SDF levels (their 95% confidence intervals and p value) to define the relative risk in predicting the abnormal standard semen parameters in study group with respect to the SDF level were calculated.

## 3. Results

**Figure 1:**
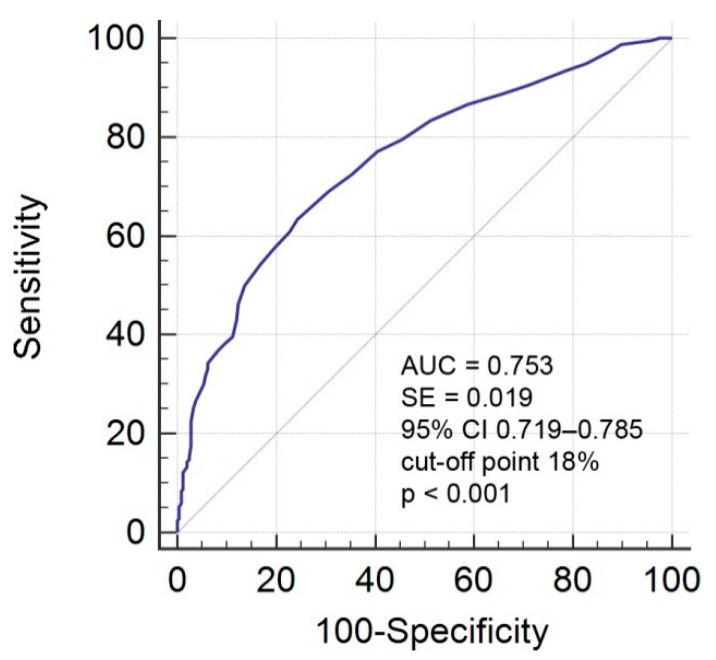
ROC curve analysis for SDF. Criterion variables are normal and abnormal standard semen parameters. AUC—area under the curve; ROC—receiver operating characteristic; SDF—sperm DNA fragmentation; *p* ≤ 0.05—statistical significance between obtained AUC vs. AUC = 0.5; CI 95%—95% confidence interval. The level of AUC was as follows: >0.7–0.8—satisfactory predictive value.

**Figure 2:**
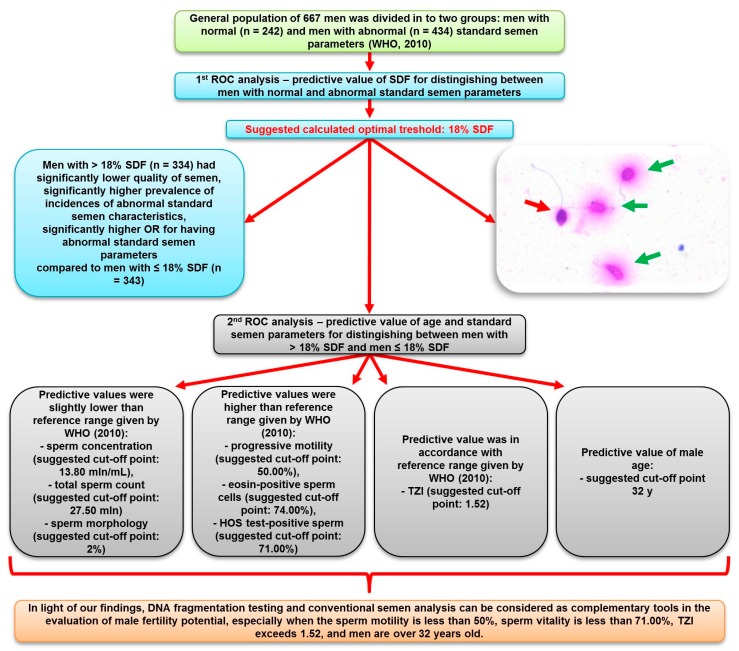
Scheme illustrating strategy developed in study analyses. ROC—receiver operating characteristic; SDF—sperm DNA fragmentation; OR—odds ratio; TZI—teratozoospermia index. Insert—visualization of sperm chromatin dispersion measured by Halo Sperm test. Spermatozoa with a big halo were considered as cells without SDF (green arrows), while those with a small halo were considered as cells with SDF (red arrow) (details in text).

The first performed ROC analysis provided suggested an optimal satisfactory threshold of 18% SDF (AUC = 0.753) to distinguish between men with abnormal and normal standard semen parameters (Figure 1 and Figure 2). Based on this ROC analysis, study groups were divided into two groups: men with > 18% SDF (n = 334) and men with ≤ 18% SDF (n = 343). A comparison of the groups showed statistically significant differences (*p* < 0.05) in evaluated parameters except for semen volume (Table 1). Men with >18% SDF were significantly older (median: 33.00 y vs. 31.00 y) and had a lower sperm concentration (median: 14.60 mln/mL vs. 25.70 mln/mL), lower total number of sperm cells (median: 53.32 mln vs. 75.62 mln), lower number of spermatozoa with normal morphology (median: 1.00% vs. 4.00%), lower number of sperm cells with progressive motility (median: 39.00% vs. 61.00%), fewer eosin-negative (live) spermatozoa (median: 70.50% vs. 81.00%), and fewer HOS test-positive (live) spermatozoa (median: 70.00% vs. 80.00%). In addition, they had significantly higher TZI (median: 1.63 vs. 1.50) and a higher concentration of peroxidase-positive cells (median: 0.25 mln/mL vs. 0.12 mln/mL) compared to men with ≤ 18% SDF. 

Moreover, men with > 18% SDF presented a significantly higher prevalence of abnormal standard semen parameters than men with SDF ≤ 18% (incidence of abnormal standard semen parameters: 82.34% vs. 46.36%, respectively) (Table 2). Additionally, men with > 18% SDF had a significantly higher OR for having abnormal standard semen parameters (OR: 5.394) than men with ≤ 18% SDF (Table 3). 

The second ROC analysis provided information about the predictive value of age and standard semen analysis for sperm DNA fragmentation (Figure 2). The calculated threshold value of age (32 y), sperm concentration (13.80 mln/mL), total number of sperm cells (27.75 mln), sperm morphology (2.00%), TZI (1.52), progressive motility (50.00%), eosin-negative sperm cells (74.00%) and HOS test-positive sperm cells (71.00%) had predictive value for distinguishing between men with > 18% SDF and men with ≤ 18% SDF (Table 4); however, semen volume and concentration of peroxidase-positive cells had no predictive value. 

An evaluation of the rank Spearman correlation revealed that SDF was positively correlated with male age (r_s_ = 0.211) and TZI (r_s_ = 0.339), but was negatively correlated with sperm concentration (r_s_ = −0.289), total number of spermatozoa (r_s_ = −0.255), sperm morphology (r_s_ = −0.457), sperm progressive motility (r_s_ = −0.524), and eosin-negative and HOS-test reactive sperm cells (r_s_ = −0.524 and r_s_ = −0.537, respectively) (Table 5).

## 4. Discussion

Generally, > 30% sperm cells with DNA damage is considered to be a cut-off point for a high risk of infertility [25,28,32,33,34,35]. However, in our study, ROC analysis of SDF to distinguish between men with normal and abnormal standard semen parameters showed that the suggested optimal threshold was 18% SDF. Moreover, using our calculated cut-off point (18% SDF), significant differences in the age of men and a significant decrease in semen quality were noted. In addition, the prevalence of abnormal standard semen parameters was higher in men with SDF > 18%, and these men had a five-fold higher OR for abnormal conventional semen parameters. Furthermore, Spearman correlation coefficient rank analysis showed significant associations between conventional semen parameters and sperm DNA damage.

It should be emphasized that our obtained findings could have clinical utility. The suggested threshold of 18% SDF was lower than the reference value given by the Halosperm G2 kit manufacturer (30% SDF). Based on our findings, it seems that 30% SDF may be unsatisfactory for discriminating men with normal fertility from those with reduced fertility potential. This suggestion is partly consistent with the studies of other authors [25,34,36,37,38,39,40]. Bungum et al. [25] showed that in the range of 0–20% sperm DNA fragmentation, the chance of a spontaneous pregnancy was constant. Moreover, Majzoub et al. [36] reported that the mean of SDF for fertile subjects was 15.68 ± 0.92% (vs. infertile 27.60 ± 1.02%). In turn, Wiweko and Utami [37] demonstrated that fertile men had 19.9% SDF (vs. infertile 29.9% SDF). Additionally, other researchers [34,38,39,40] considered that 0–15% SDF is related to a high fertility potential, while 16–30% and >30% correlated with moderate and low fertility potentials, respectively.

Our second ROC evaluation revealed the predictive value of age and standard semen parameters for distinguishing between men with > 18% SDF and ≤ 18% SDF. Importantly, the cut-off points were as follows: sperm concentration 13.80 mln/mL, total sperm count 27.50 mln, and sperm morphology 2%. Our findings suggest that with the above values, which are slightly lower than the reference range given by WHO [29], we can expect better quality sperm DNA. On the other hand, the cut-off point for progressive motility was 50.00%, eosin-positive sperm cells was 74.00%, and HOS test-positive sperm cells was 71.00%. This means that we can expect better quality sperm DNA when values of sperm progressive motility and vitality are significantly higher than the WHO criteria. In turn, only the cut-off point for TZI (1.52) was in accordance with data reported by Menkveld et al. [41], in which an increase above this value may result in a decrease in sperm DNA quality. This result concerning the predictive value of male age for sperm DNA damage was unexpected. The study suggested that an age above 32 years can correlate with the deterioration of the quality of sperm chromatin. This result is particularly interesting, because most authors show that a decline in sperm DNA quality usually occurs after the age of 40 and sometimes after 35, which is commonly classified as advanced paternal age [10,42,43,44].

## 5. Study Limitations

Some limitations of our study must be addressed. Firstly, the method we used to reveal SDF was the SCD, and we have to highlight that this method has some known disadvantages, such as the indirect assessment of sperm DNA and the susceptibility to subjective assessment of sperm DNA dispersion, where there is a risk of over-interpretation of the results [45,46,47]. As presented by Javed et al. [45] and Ribas-Maynou et al. [47], these methods directly assessed with double-strand DNA breaks like alkaline comet test, TUNEL, or SCSA, have grater clinical utility for distinguishing between fertile and infertile patients than SCD. On the other hand, those same authors admitted that an SCD test also has an important clinical utility [45,47]. Moreover, this method had some advantages: it is simple to perform, precise, highly reproducible, inexpensive, and advanced laboratory equipment is not required [46]. Furthermore, some authors [11,12,45,46] indicated that the complementary assessment of male fertility potential, including standard semen parameters and the SCD method, is justified and may provide clinically significant data about sperm fertilizing ability. 

It is also worth mentioning that the clinically useful threshold of SDF is difficult to estimate [24,25,26,27,28,48]. In our study, the cut-off point of SDF for distinguishing between men with normal and abnormal standard semen parameters was 18%, not 30%, as previously suggested by the manufacturer of the Halo Sperm test. It is possible that if we limited the group of men to those with proven fertility and to those with isolated male fertility problems, the threshold value of SDF based on ROC analysis would be different.

## 6. Conclusions 

In light of our findings, we can conclude that men with >18% SDF have a higher risk for abnormal standard semen parameters, while age and obtained standard semen parameters have a predictive value for SDF. Our statistical data indicate association between SDF and parameters of basic semen analysis; however, it is possible that men with normal standard semen parameters may have reduced fertility potential due to diminished sperm chromatin integrity. Therefore, DNA fragmentation testing and conventional semen analysis can be considered as complementary tools in the evaluation of male fertility potential.

## Figures and Tables

**Table 1:** Descriptive statistics and comparison of age and standard semen parameters in men with >18% SDF and men with ≤18% SDF.

Parameters	Total	Men with >18% SDF	Men with ≤18% SDF
	n	n	n
	Median (Range)	Median (Range)	Median (Range)
	Mean ± SD	Mean ± SD	Mean ± SD
Age (y)	n = 667	n = 334	n = 343
	32.00 (19.00–54.00)	33.00 (21.00–54.00) **	31.00 (19.00–49.00)
	32.20 ± 5.80	33.23 ± 5.76	31.19 ± 5.66
Semen volume (mL)	n = 677	n = 334	n = 434
	3.00 (0.50–11.50)	3.25 (0.50–11.50)	3.00 (0.50–10.00)
	3.59 ± 1.69	3.64 ± 1.81	3.54 ± 1.57
Sperm concentration (×10^6^/mL)	n = 677	n = 334	n = 343
	19.92 (0.05–283.00)	14.60 (0.05–166.00) **	25.70 (0.25–283.00)
	28.04 ± 30.37	22.44 ± 25.21	33.49 ± 33.83
Total number of spermatozoa (×10^6^)	n = 677	n = 334	n = 343
	66.00 (0.25–672.00)	53.32 (0.25–672.00) **	75.62 (0.50–660.25)
	92.90 ± 95.45	75.48 ± 83.86	109.87 ± 102.83
Morphologically normal spermatozoa (%)	n = 677	n = 334	n = 343
	2.00 (0.00–15.00)	1.00 (0.00–13.00) **	4.00 (0.00–15.00)
	3.10 ± 3.07	1.86 ± 2.47	4.31 ± 3.12
TZI	n = 677	n = 334	n = 343
	1.55 (1.13–2.58)	1.63 (1.20–2.58) **	1.50 (1.13–2.46)
	1.60 ± 0.22	1.67 ± 0.24	1.62 ± 0.19
Sperm progressive motility (%)	n = 677	n = 334	n = 343
	51.00 (0.00–89.00)	39.00 (0.00–85.00) **	61.00 (2.00–89.00)
	47.68 ± 21.98	38.16 ± 21.39	56.94 ± 18.31
Eosin-negative spermatozoa—live cells (%)	n = 677	n = 334	n = 343
	77.00 (0.00–96.00)	70.50 (0.00–94.00) **	81.00 (14.00–96.00)
	72.73 ± 17.02	65.80 ± 19.11	79.48 ± 11.17
HOS test-positive spermatozoa—live cells (%)	n = 615	n = 288	n = 327
	76.00 (0.00–94.00)	70.00 (0.00–91.00) **	80.00 (12.00–94.00)
	71.76 ± 16.97	64.77 ± 19.05	77.91 ± 11.91
Peroxidase-positive cells (mln/mL)	n = 677	n = 334	n = 343
	0.20 (0.00–27.00)	0.25 (0.00–10.25) *	0.12 (0.00–27.00)
	0.49 ± 1.45	0.53 ± 1.21	0.24 ± 0.00

HOS test—hypo-osmotic swelling test; n—number of subjects; SD—standard deviation; SDF—sperm DNA fragmentation; TZI—teratozoospermia index. * Significant differences between men with ≤18% SDF at *p* < 0.010; ** Significant differences between men with ≤18% SDF at *p* < 0.001, Mann–Whitney U test.

**Table 2:** Prevalence of normal and abnormal standard semen parameters in men with >18% SDF and ≤18% SDF.

Group	Standard Semen Parameters	
	Normal N (%)	Abnormal ^&^ N (%)
**Men with > 18% SDF (n = 334)**	59 (17.66) **	275 (82.34) **
**Men with ≤ 18% SDF (n = 343)**	184 (53.64)	159 (46.36)

^&^ At least one abnormal standard semen parameter according to the WHO [29] (details in Subject section). n—number of subjects. ** Significant differences between men with ≤18% SDF at *p* < 0.001, chi^2^ test. SDF—sperm DNA fragmentation.

**Table 3:** Odds ratio (OR) for abnormal standard semen parameters in men with >18% SDF (n = 334) compared to men with ≤18% SDF (n = 343).

Semen Category	Men with >18% SDF N (%)	Men with ≤18% SDF N (%)	OR (95%CI)
Abnormal standard semen parameters ^&^	275 (82.34)	159 (46.36)	5.394 (3.7922–7.6720) **

^&^ At least one abnormal standard semen parameter WHO [29] (details in Subject section). n—number of subjects. ** Statistical significance at *p* < 0.001; 95% CI—95% confidence interval. SDF—sperm DNA fragmentation.

**Table 4:** ROC curve analysis for age and standard semen parameters. Criterion variable is SDF level >18%.

Parameter	AUC	SE	CI 95%	Suggested Optimal Cut-Off Point
Age (y)	0.601 **	0.021	0.563–0.638	32.00
Semen volume (mL)	0.506	0.022	0.468–0.545	6.00
Sperm concentration (×10^6^/mL)	0.641 **	0.021	0.603–0.677	13.80
Total number of spermatozoa (×10^6^)	0.625 **	0.021	0.587–0.661	27.75
Morphologically normal spermatozoa (%)	0.740 **	0.018	0.705–0.772	2.00
TZI	0.677 **	0.020	0.641–0.713	1.52
Sperm progressive motility (%)	0.746 **	0.018	0.711–0.778	50.00
Eosine-negative spermatozoa—live cells (%)	0.743 **	0.018	0.708–0.775	74.00
HOS test-positive spermatozoa—live cells (%)	0.743 **	0.019	0.706–0.777	71.00
Peroxidase-positive cells (mln/mL)	0.567	0.021	0.529–0.605	0.00

AUC—area under the curve; ** Statistical significance with AUC = 0.5 at *p* < 0.001; CI 95%—95% confidence interval; HOS test—hypo-osmotic swelling test; ROC—receiver operating characteristic; SDF—sperm DNA fragmentation; TZ—teratozoospermia index. The levels of AUC were as follows: 0.9–1.0—excellent predictive value; >0.8–0.9—good predictive value; >0.7–0.8—satisfactory predictive value; >0.6–0.7—moderate predictive value; and 0.5–0.6—insufficient predictive value.

**Table 5:** Rank Spearman correlations (r_s_) between human sperm chromatin fragmentation (SDF), male age and standard semen parameters (n = 676).

Parameters	r_s_
Age (y)	0.211 *p* < 0.001
Semen volume (mL)	−0.010 *p* = 0.794
Sperm concentration (×10^6^/mL)	−0.289 *p* < 0.001
Total number of spermatozoa (×10^6^)	−0.255 *p* < 0.001
Morphologically normal spermatozoa (%)	−0.457 *p* < 0.001
TZI	0.339 *p* < 0.001
Sperm progressive motility (%)	−0.524 *p* < 0.001
Eosin-negative spermatozoa—live cells (%)	−0.524 *p* < 0.001
HOS test-positive spermatozoa—live cells (%)	−0.537 *p* < 0.001
Peroxidase-positive cells (mln/mL)	0.125 *p* = 0.001

The interpretation of r_s_ value: < 0.2 lack of linear dependence; 0.2–0.4—weak dependence; >0.4–0.7—moderate dependence; >0.7–0.9—strong dependence; and > 0.9—very strong dependence. n—number of subjects, statistical significance at *p* < 0.05; HOS test—hypo-osmotic swelling test, TZI—teratozoospermia index.
